# Screening for ADHD Symptoms among Criminal Offenders: Exploring the Association with Clinical Features

**DOI:** 10.3390/healthcare10020180

**Published:** 2022-01-18

**Authors:** Enrico Capuzzi, Martina Capellazzi, Alice Caldiroli, Francesca Cova, Anna Maria Auxilia, Paola Rubelli, Ilaria Tagliabue, Francesco Giuseppe Zanvit, Gianluca Peschi, Massimiliano Buoli, Massimo Clerici

**Affiliations:** 1 Psychiatric Department, Azienda Socio Sanitaria Territoriale Monza, 20900 Monza, Italy; alyscaldi@gmail.com (A.C.); f.cova@asst-monza.it (F.C.); paolarubelli@yahoo.it (P.R.); massimo.clerici@unimib.it (M.C.); 2 Department of Medicine and Surgery, University of Milano Bicocca, 20900 Monza, Italy; martina.capellazzi@gmail.com (M.C.); annam.auxilia@gmail.com (A.M.A.); ilariatagliabue11@gmail.com (I.T.); francesco.zanvit@gmail.com (F.G.Z.); 3 Department of Social and Health Care, Azienda Socio Sanitaria Territoriale Monza, 20900 Monza, Italy; g.peschi@asst-monza.it; 4 Department of Neurosciences and Mental Health, Fondazione IRCCS Ca’Granda Ospedale Maggiore Policlinico, 20122 Milan, Italy; massimiliano.buoli@hotmail.it; 5 Department of Pathophysiology and Transplantation, University of Milan, 20122 Milan, Italy

**Keywords:** attention-deficit/hyperactivity disorder, prison, comorbid, childhood trauma

## Abstract

Background: Attention-deficit/hyperactivity disorder (ADHD) is a disabling disorder. High rates of ADHD have been consistently reported among prisoners. The main objectives were (1) to estimate the prevalence of ADHD symptoms in a sample of male inmates and (2) to investigate the relationship between ADHD symptoms and socio-demographic/clinical features. According to the high prevalence of childhood trauma among inmates, we assessed whether exposition to childhood trauma can be related to the presence of ADHD symptoms. Methods: A total of 159 male prisoners admitted to Monza prison between January 2020 and June 2021 were included. Both Wender Utah ADHD rating scale and adult ADHD self-report scale were administered to assess ADHD symptoms. Moreover, inmates completed the childhood trauma questionnaire. Results: Data were available for 108 inmates. Thirty-five prisoners (32.4%) were found on screening to meet the criteria for symptoms of ADHD. Cocaine use disorder, prescription of mood stabilizers and a history of emotional abuse significantly increased the likelihood of having clinically significant ADHD symptoms. Furthermore, patients who experienced physical neglect resulted in meeting the criteria for ADHD symptoms. Conclusions: ADHD symptoms are widespread among inmates and are associated with specific risk factors. Screening for ADHD should be done to provide appropriate intervention strategies.

## 1. Introduction

According to the Diagnostic and Statistical Manual of Mental Disorders, Fifth Edition (DSM-5), Attention-deficit/hyperactivity disorder (ADHD) is defined as a persistent pattern of inattention and/or hyperactivity/impulsivity that interferes with functioning or development. A level of impairment in a minimum of two areas of life needs to be manifest for at least six months to make diagnosis of ADHD [1]. Moreover, the American Psychiatric Association lists three subtypes of ADHD in the DSM-5. These are a predominantly inattentive type, a predominantly hyperactive–impulsive type, and a combined type for those with both hyperactive–impulsive and inattentive symptoms [1]. Symptoms of ADHD typically first occur between the ages of three and six years old and persist in adulthood in 40–60% of cases [2]. Therefore, ADHD is a prevalent disorder, affecting 0.6–7.3% of the general adult population [3]. However, higher rates of ADHD were reported among criminal offenders compared to the general population. Indeed, even though most studies may suffer from major methodological shortcomings [4], the prevalence of adult ADHD in prison inmates is generally detected to be 10 times higher than in general population. According to the meta-analysis by Young et al. [5], the estimated prevalence of ADHD among incarcerated populations is 25.5% (more specifically, 26.2% in adult prison populations and 30.1% in youth prison populations). Large gaps were reported between countries since the ADHD prevalence ranged from 6.6% in Brazil to 65.2% in Sweden. However, when grouped into three regions (i.e., North America, Europe, and other countries), these differences were not significant [5]. In our knowledge, no Italian studies were performed to explore the prevalence of ADHD symptoms in adult inmates On the other hand, studies exploring the relationship between ADHD and crime provide additional evidence that patients with ADHD are not only more likely to be involved in judicial system but that they are also younger at first conviction and at first arrest [6]. It is noteworthy that around half of children with ADHD will suffer from a conduct disorder, which, in turn, is associated with antisocial personality disorder in adulthood [7]. A severe impulsivity combined with inattention and hyperactivity seems to be the most predisposing factor for the subsequent occurrence of antisocial behaviors and recidivism [8]. Nevertheless, it is well established that as many as 80% of individuals with ADHD have several psychiatric (e.g., anxiety, mood disorders, and personality disorders) and medical comorbidities [9,10]. Among incarcerated adults with ADHD, there is therefore a high risk of misdiagnosis and inappropriate treatment. In particular, most studies reported a high proportion of adult offenders with a primary diagnosis of personality disorder but with childhood-onset ADHD [11]. In addition, substance use disorders are generally concomitant with adult ADHD, particularly when there is a comorbid antisocial behavior [7]. The presence of ADHD, in turn, may represent an important risk factor for both suicidal ideation and suicidal behaviors among prisoners, especially when the disorder is in comorbidity with substance use disorders [12]. Moreover, it should be taken into account that exposure to early stressful life events, e.g., childhood trauma, increases vulnerability to ADHD as well as the persistence of the disorder into adulthood and the onset of comorbid mental disorders [13]. About 20–50% of children with a history of childhood trauma have clinically significant ADHD symptoms [14]. Even though a strong relationship between the presence of ADHD symptoms and childhood trauma was suggested, it is still not clear whether children who show symptoms of ADHD are more likely to be abused and neglected or whether experiencing childhood trauma may increase the likelihood of the development of ADHD symptoms [15]. Nevertheless, children with ADHD are at elevated risk of maltreatment and to develop a subsequent antisocial personality disorder [16]. Gender differences emerged with respect to the type of childhood trauma. Emotional abuse and neglect seem to be more frequent among men and women with ADHD as compared to controls, whilst females with ADHD generally report sexual abuse and physical neglect [16]. What has not been examined till now is whether prisoners with ADHD are more likely to report a childhood trauma. It is noteworthy that prisoners have high prevalence of childhood trauma, including abuse and neglect [17]. In the United States, 1 in 6 state male inmates reported being physically or sexually abused in childhood or adolescence, and many more witnessed interpersonal violence. Furthermore, more than half of male inmates reported experiencing childhood physical trauma. By contrast, sexual trauma in childhood is less common than physical trauma among men prisoners [18]. Even though prevalence estimates varied substantially across studies for all types of abuse and neglect, according to a recent meta-analysis, half of people in prisons may have experienced some trauma in childhood [19]. Nevertheless, some authors elaborated on the theory of the “cycle of violence”, whereby children exposed to violence may present a higher risk to become violent during adulthood, as well as to present an increased risk of later suicide attempts, aggressive behaviour, and mental disorders [20]. Indeed, experiencing physical, sexual, or emotional abuse during childhood is known to have predictable immediate and distal impacts on personality development.

Even though there was increasing sensitivity in the recognition and management of ADHD in the community, few studies focused on this topic with regard to subjects hosted in detention centers. Nevertheless, the relationship between ADHD and other psychiatric comorbidities, substance use disorders, and childhood trauma is still poorly studied in prison populations. With our study, we firstly aimed to investigate the prevalence of ADHD symptoms among a sample of adult male prisoners. Secondly, we aimed to find potential predictors of clinically significant ADHD symptoms for incarcerated male subjects with a specific focus on the different types of coexisting mental disorders (including substance use disorders) and childhood trauma. As discussed above, there is an urgent need to identify adult offenders with ADHD symptoms and to intervene proactively. Indeed, we hypothesized that ADHD is prevalent among prisoners and that subjects with ADHD symptoms have more comorbid mental disorders and substance misuse compared to the ones without ADHD symptoms. Moreover, we expected that one or more types of childhood trauma might better discriminate between the ADHD and non-ADHD groups, thus increasing knowledge regarding this condition. These aspects are of current interest because prisoners with ADHD symptoms are frequently undiagnosed, and they may be treated for a comorbid psychiatric condition instead of their underlying ADHD [21,22,23].

## 2. Materials and Methods

### 2.1. Setting and Study Design

This is a cross-sectional study of consecutive newly arrived inmates admitted to Monza prison (Psychiatric Department of ASST, Monza, Italy), between January 2020 and June 2021. At present, the prison houses only male offenders. Newly arrived prisoners eligible for this study were on remand or sentenced, admitted in the penitentiary for the first time or formerly incarcerated, aged between 18 and 65 years, and they expressed a willingness to participate in the study. We excluded prisoners unable to speak or read Italian or with too severe cognitive impairment, learning disabilities, and psychiatric symptoms to complete the study. Moreover, participants with any missing data were excluded. A specifically trained team of psychiatrists and psychologists conducted a clinical interview within the first 72 h after the incarceration. Indeed, in most adult prisons newly arrived offenders are subject to a reception screen upon admission aimed to review their overall mental health problems, including ADHD, as well as detecting the risk of self-injurious behaviours and suicidality [23].

### 2.2. Measures

All data were anonymously collected and registered in an appropriate dataset, not allowing patients’ identification. Self-reported measures included age, nationality (Italian versus other), partnership (single/divorced/widowed versus partner/married), education level (low-medium versus high), unemployment (yes versus no), and previous incarceration (yes versus no). Moreover, participants were asked about history of nonsuicidal self-injuries (NSSIs) and attempted suicide in and out of prison. NSSI is defined as the deliberate, direct, socially unacceptable destruction or alteration of body tissue (i.e., cutting, burning, hitting oneself, scratching wrists or arms, banging one’s head against the wall). On the other hand, we considered as ‘suicide attempts’ only self-injuries of people willing to die [24]. Although some studies have shown a significant overlap between NSSI and suicide attempts, there is an urgent need to identify what type of self-harm might be more associated with ADHD and, consequently, with emotional dysregulation [25]. Prisoners were also asked if they were referred to outpatient child and adolescent psychiatry services or outpatient adult mental health care.

The prescription of psychotropic medications was defined by the use of at least one of the following drugs: antipsychotics, mood stabilizers (lithium and anticonvulsant agents), and antidepressants, as well as anxiolytics or hypnotics. We included psychotropic medications prescribed both before imprisonment (according to medical records or self-reported data) and within the first 72 h after incarceration. In this regard, psychotropic medications could have been prescribed by either psychiatrists or other medical practitioners working in the prison. Information about comorbid medical conditions (hypertension, diabetes, cardiovascular diseases, cancer, liver and lung diseases, or other disorders) was acquired according to medical records or patients’ self-report.

ADHD symptoms were assessed by asking adults to complete both the Wender Utah ADHD rating scale (WURS) and adult ADHD self-report scale (ASRS). The 25-item version of the WURS was used to retrospectively evaluate ADHD-related symptoms in childhood [26]. The adults were asked to rate the presence of childhood behaviour described by the items on a scale from 0 (“not at all or very slightly”) to 4 (“very much”). The items are chosen from the original 61-item version of WURS for their ability to discriminate between ADHD and controls. WURS-25 provides a total sum score (range 0–100), and, according to the originators, a cut-off score of 46 or higher correctly identifies 86% of the patients with ADHD [27]. The ASRS is a self-report, Diagnostic and Statistical Manual of Mental Disorders-IV-based measure, with 18 questions regarding current symptoms of adult ADHD [27]. The instrument includes two parts: Part A (6 questions) and Part B (12 questions). The six items of part A, four concerning attention-deficit symptoms and two concerning hyperactivity/impulsivity symptoms, showed great sensitivity and can be used as a separate screening tool, also known as the ASRS-V1.1 short form. Respondents are asked to rate each item, using a five-point response scale ranging from never (0) to very often (4). A patient is considered to be positive on the ASRS-v1.1 if at least four items of the part A overcome the threshold of clinically significant ADHD symptoms. However, a positive test cannot be considered sufficient to make a diagnosis of adult ADHD, but it does indicate that further diagnostic investigation is required [28,29]. The WURS-25 has been validated by Italian investigators [25]. However, despite the lack of an Italian validation of ASRS in adult population [30], this latter is a standard tool in clinical practice. In the light of these considerations, participants who met the criteria for clinically significant ADHD symptoms on both scales were considered to be suffering from the disorder [31,32,33].

Substance use and other mental disorders were assessed according to the diagnostic criteria of the DSM-5. Drug-specific diagnoses for the following substances were included: alcohol, cannabis, cocaine, heroin, hallucinogens, ecstasy, ketamine, inhalants, prescription opioids, sedatives or tranquilizers, and stimulants. Each DSM-5 substance use disorder diagnosis required fulfilling two or more of the 11 criteria in the 12 months preceding the interview [34].

History of childhood trauma was evaluated by the 34-item CTQ [35]. The CTQ yields scores for childhood emotional, physical and sexual abuse, and emotional and physical neglect. The reliability and consistency of this tool have been already demonstrated [36]. A history of specific trauma types (yes versus no) was defined by the presence of at least moderate childhood victimization similarly to determinations by previous investigation [37].

### 2.3. Data Analysis

Descriptive analyses of included variables were performed for the total sample: mean and standard deviation (SD) for quantitative variables and frequency and percentage for qualitative ones. The total sample was divided into ADHD-screened positive (ADHD-P) and ADHD-screened negative (ADHD-N) groups according to the cut-off levels for WURS-25 and ASRS-V 1.1, respectively. First, we carried out univariate analysis in order to detect statistically significant differences in these two groups. The normal distribution of quantitative variables was verified by using Shapiro–Wilk’s test. Age was compared between groups by independent sample T test. The groups were compared for qualitative variables by chi-square tests. Finally, all variables from the univariate analysis with *p* < 0.05, together with age and previous incarceration, were entered as independent variables into a logistic regression model with ADHD-P as the dependent variable. Adjusted odds ratios (aOR), together with their 95% confidence intervals (CI), were reported.

Analysis was conducted using Stata Version 13.1 SE (College Station, TX, USA: StataCorp LP).

## 3. Results

**Figure 1:**
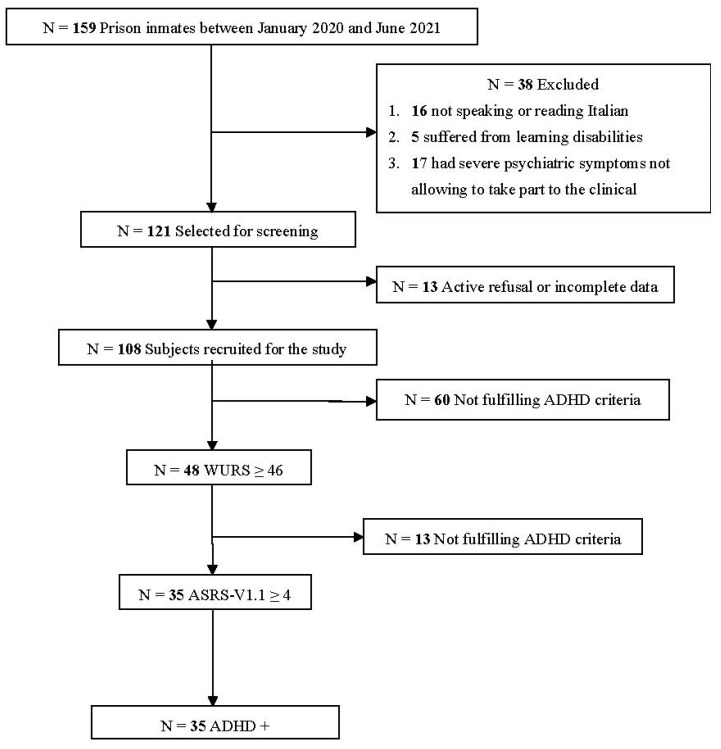
Flowchart of ADHD screening procedures. ADHD = attention-deficit/hyperactivity disorder; ASRS-V 1.1 = the adult ADHD self-report scale v1.1; WURS = Wender Utah rating scale.

Our original sample consisted of 159 inmates. Among these, 38 were excluded because they did not meet inclusion criteria: 16 did not speak or read Italian; 5 suffered from learning disabilities; 17 individuals had severe psychiatric symptoms not allowing them to take part to clinical interview. Moreover, 13 were excluded in view of active refusal or incomplete data. Therefore, data were available for 108 male inmates. A flow chart of selected subjects is reported in Figure 1.

According to the cut-off levels for both WURS-25 and ASRS-V 1.1, thirty-five prisoners (32.4%) were considered ADHD-P. The majority of our sample (57.0%) was previously incarcerated. Personality disorders (45.5%) were the most common diagnosis, followed by psychotic disorders (11.2%) and depressive and adjustment disorders (11.1%). The highest 12-month prevalence rates of substance use disorders were for cocaine (73.1%), cannabis (60.2%), and alcohol (33.3%). Unlike NSSI (25.0%), most individuals reported to have attempted suicide out of prison (9.3%). Anxiolytics or hypnotics (67.6%) and antipsychotics (35.2%) were the most commonly prescribed psychotropic medications in the whole sample. The most-experienced childhood trauma was emotional (20.4%) and physical (19.4%) abuse (Table 1).

Age was statistically lower in the ADHD-P group. Prisoners with a diagnosis of personality disorder, as well as of cocaine use disorder, were more likely to be ADHD-P. ADHD-P subjects had higher prevalence of NSSIs in prison. The ADHD-P group reported a higher rate of referral to outpatient child and adolescent psychiatry services as compared with the ADHD-N group. Moreover, a higher rate of the prescription of mood stabilisers and anxiolytics or hypnotics was found among ADHD-P prisoners. With regards to childhood trauma, a history of emotional abuse and physical neglect was associated with the presence of ADHD-P.

Finally, after controlling for age and previous incarceration, we found an association between the presence of ADHD-P and cocaine use disorder (aOR = 5.60, *p* = 0.020) and prescription of mood stabilisers (aOR = 5.14, *p* = 0.003), as well as a history of emotional abuse (aOR = 3.65, *p* = 0.039) (Table 2).

## 4. Discussion

### 4.1. Main Findings

Apart from a pilot study conducted on a sample of 59 Italian inmates [38], to the best of our knowledge, this is the first study aimed to examine the prevalence of ADHD symptoms and clinical correlates in newly arrived adult inmates of an Italian prison. According to our findings, almost 1 out of 3 prisoners were found to meet the criteria for possible ADHD using both WURS and ASRS. Even though estimates of the prevalence of ADHD symptoms vary largely in the literature in relation to different information sources, diagnostic criteria, and sample characteristics [39], our figure is consistent with two previous studies reporting a considerable percentage of ADHD diagnoses in prison compared with the general population. Particularly, Einarsson et al. [7] found that half of male prisoners (*n* = 90) who were assessed within 10 days of admission to the detention centre met the criteria for ADHD in childhood, and 60% of those were either fully symptomatic or in partial remission of their symptoms. On the other hand, a study conducted on a sample of 194 adult male longer-term prison inmates by Ginsberg et al. [40] reported an estimated prevalence of 40% of adult ADHD. Consequently, it is likely that a large percentage of inmates affected by ADHD may be unrecognized and untreated [39]. Indeed, screening is mostly the first step of the diagnostic process and not a substitute for clinical diagnosis, which is typically considered prominent in the identification of ADHD. Nevertheless, a meta-analysis of 42 prisons, based on international data resulting from symptom-based diagnostic interviews, indicated that 25.5% of the prison population overall met the diagnostic criteria for ADHD [5], with no significant gender difference [41].

Beyond the high prevalence of ADHD-P within our sample of male prisoners, we found three main results. First of all, we observed that ADHD-P group was more likely to have cocaine use disorder than ADHD-N one. Different hypotheses have been proposed to clarify the strong relationship between substance use disorder and ADHD. Familial studies confirmed a shared genetic substrate between ADHD and substance use disorder [42]. Moreover, known risk factors for substance use disorder such as “sensation-seeking” behaviour and impulsivity traits are shared with ADHD [43]. Nevertheless, many patients may use addictive drugs as a method of “self-medication” to suppress ADHD symptoms. Indeed, ADHD individuals are inclined to use stimulants paradoxically in order to reduce mental and physical restlessness, emotional moodiness, and inattentiveness as a result of dopamine dysregulation [44]. It is also noteworthy that dopamine neurotransmission is involved both in ADHD and substance use disorder [45]. In this regard, cocaine acts on different brain circuits, including those implicated in reward (accumbens and ventral pallidum), working memory (hippocampus and amygdala), control (cingulated gyrus and prefrontal cortex), and volition (orbitofrontal and subcallosal cortices), which are compromised in ADHD patients and are therefore the primary targets of pharmacological treatments [46,47]. However, with regard to legal problems, ADHD patients abusing alcohol, cocaine, and other stimulants show a higher frequency of violent behaviours as well as antisocial conduct [48]. Even though substance use in subjects with ADHD can be considered a way to alleviate symptoms, the combined use of stimulants and alcohol may have the opposite result, leading to aggressiveness, behavioural disturbances, and legal consequences [49]. Consistently with the literature on ADHD [50], we found that the substances most-used by our patients were cocaine, THC, and alcohol. However, only cocaine use disorder has a statistically significant relationship with ADHD-P. Of note, we cannot exclude that most prisoners were poly-abusers and that the type of abused substance depends on different variables, including history of a conduct disorder and the age-related clinical presentation of ADHD [51].

Second, we found that ADHD-P prisoners were more likely to receive the prescription of a mood stabiliser than ADHD-N prisoners. ADHD can present with irritability, emotional dysregulation, mood lability, low self-esteem, low frustration-tolerance, and sleep problems, favouring misdiagnosis with other mental conditions such as personality disorders and mood disorders [31]. Nevertheless, according to univariate analysis, we found a higher rate of personality disorders among ADHD-P prisoners compared with the counterpart. However, compared with other offenders, prisoners with ADHD are more likely to exhibit more mood swings and critical incidents within a detention environment. Indeed, their behavioural problems can worsen once they are incarcerated because their impulsivity may result in an increased level of inconsistent and unacceptable behaviour, including verbal and physical aggression [9]. On the other hand, and according to our results, we cannot completely exclude that cocaine use disorder may cause significant changes in mood, increasing the risk of impulsive behaviour, as well mask different symptoms of ADHD, therefore representing a confounding factor in the correct recognition of ADHD and the definition of an effective strategy of treatment [49]. It should be noted that, even though several studies reported that second-line treatment options such as mood stabilizers are effective in reducing mood shifts and aggressive behaviours [52], the opportunity for access to first-line pharmacological treatment for adults with ADHD (e.g., atomoxetine) is extremely complicated and unlikely in prison [22]. Despite less robust association than results statistically significant in the final logistic regression model, we found a more frequent prescription of anxiolytics or hypnotics in ADHD-P prisoners versus ADHD-N ones. However, when treatments are prescribed only for symptom control without any significant effect on the long-term course of ADHD, the disorder will continue to interfere with individuals’ lives as a result of an increased risk of aggression and violence towards others, self-harm, and suicide both during incarceration and upon release from prison [10]. Indeed, untreated ADHD may directly contribute to high social dysfunction and disability as a result of cognitive impairment and comorbid disorders. Therefore, given the high risk of comorbid mental disorders and the increased risk of self-harm or suicide in the first weeks of detention among adult offenders with ADHD, it appears essential to identify prisoners at risk of aggression, violence, self-harm, and suicide who might benefit from treatment for ADHD [21,23].

Third, childhood trauma was significantly related to the presence of ADHD-P. To the best of our knowledge, this is the first study exploring the possible connection between childhood trauma subtypes and ADHD among prison inmates. Even though multiple factors contribute to the etiology of ADHD, a growing body of research has hypothesized a relationship between ADHD onset and early stressful life events, more specifically childhood trauma, in both children and adult samples. Indeed, different studies indicated that childhood trauma occurring prior to young adulthood may be more common among individuals with ADHD in comparison to non-ADHD groups, and greater levels of ADHD symptoms may be found among people who were exposed to childhood trauma compared to non-exposed subjects [53]. However, other authors reported opposite findings [54]. Of note, some longitudinal studies failed to find a causal association between child abuse/neglect and adult ADHD, after adjusting for childhood ADHD [15]. Nevertheless, the psychological sequelae of childhood trauma may include other mental conditions, including anxiety, depression, behavioural problems, aggression, dissociative symptoms, and post-traumatic stress disorder, that may be challenging to differentiate from ADHD. Particularly, the association between ADHD and post-traumatic stress disorder could be bidirectional [55]. On the other hand, it is still not clear whether children who show symptoms of ADHD are more likely to be victimized or whether having experienced a childhood trauma may increase the likelihood of developing ADHD symptoms. Indeed, children having ADHD symptoms, in particular when comorbid with conduct disorder, could be extremely prone to experience childhood trauma since they frequently manifest oppositional behaviour or because their family members in turn have impulsive disorders [15]. According to our final logistic regression model, emotional abuse was significantly related to the presence of ADHD symptoms. Some models tried to explain the association between emotional abuse and ADHD. For instance, some studies suggested that emotional abuse might be associated with the formation of negative beliefs about self and others, leading to a cognitive vulnerability to ADHD [56]. In this regard, a systematic review study by Maguire et al. (2014) showed that children exposed to emotional abuse may subsequently exhibit a wide range of negative effects, including ADHD [57]. Furthermore, emotional abuse may be significantly related to substance use disorder, which in turn is associated with changes in brain structures and enhances susceptibility to ADHD [58]. Hence, childhood trauma and substance use disorder might represent two different environmental stressors, acting as triggers of ADHD on individuals with genetic vulnerability [59]. On the other hand, we found that ADHD-P subjects had higher prevalence of NSSIs in prison. Interestingly, there is research evidence that, in survivors of childhood trauma, particularly of emotional abuse, emotional dysregulation could be a key modulator of NSSI in ADHD. Therefore, higher levels of emotional dysregulation in adult ADHD, together with childhood exposure to emotional abuse, may contribute to the complexity of clinical presentation among self-harming individuals [60].

Finally, despite less robust association than results statistically significant in the final logistic regression model, patients diagnosed with personality disorder and history of NSSIs are more frequently ADHD-P. To date, it is widely recognized that prisoners with ADHD symptoms have an increased risk of comorbid disorders. According to a meta-analysis of 18 studies including 1615 individuals with ADHD and 3128 without ADHD, adult prisoners with ADHD had a 3-time higher risk of personality disorders compared to the counterpart [61]. Nonetheless, comorbidity with personality disorders makes ADHD be characterized by more impulsivity and cognitive impairment, resulting in poor social functioning [62]. Moreover, as to date there are no data in the literature, we cannot completely exclude that other specific type of CT, including physical neglect, together with other factors, such as biological ones, may increase vulnerability to ADHD symptoms [63]. Therefore, routine inquiries about childhood trauma in cases of adult ADHD are strongly recommended, although assumptions should not be made that actual psychiatric problems are a result of abuse or neglect. Nevertheless, clinicians should be cautious with the prescription of combined treatments, given that the interventions for ADHD, post-traumatic stress disorder, and victimisation are distinct and may even be harmful if misdirected. Indeed, according to a large systematic meta-review of several adverse effects [64], the side effects of anti-ADHD medications, including anorexia, sleeplessness, and sometimes rebound agitation, may worsen in an already vulnerable individual previously exposed to trauma. Therefore, adults who have had traumatic childhood experiences may benefit from psychosocial interventions aimed to help them to elaborate unsolved childhood experiences rather than psychotropic medications designed to alleviate symptoms but with uncertain effectiveness in the long-term for this type of problem [16]. In addition, it has been reported that some decision aid prototypes might be a promising option to facilitate the shared decision-making process about treatments in adults newly diagnosed with ADHD [65]. Even though further research on decision aids is necessary before its routine introduction, this approach may be useful in helping patients to cope with ADHD symptoms.

In any case, according to best practices, all new prisoners should receive a first reception screen for ADHD. In particular it is important to review the medical history and evaluate the presence of other common mental or neurodevelopmental disorders that may overlap with ADHD—emphasising the need for a careful diagnostic assessment [39]. As a result, appropriate interventions, including pharmacological and non-pharmacological treatments, are expected to give a positive impact on the prisoner and society and could lead to reduced rates of re-offending, increased productivity, and decreased resource utilization [22].

### 4.2. Limitations

There are some important methodological limitations related to the present study. First, as this study was a cross-sectional one, we cannot definitively define whether the independent variables preceded or followed the ADHD symptoms. Secondly, the self-report scales, despite appropriate for detecting ADHD symptoms, are less reliable to make a diagnosis of the disorder compared to semi-structured interviews. Indeed, the requirement to be positive for ADHD in two different rating scales increases the reliability, but it still does not totally remove potential recall bias [31]. Therefore, the rate of ADHD-P individuals from this study consist of individuals who “resulted positive in self-report ADHD instruments” and not with a definitive ADHD diagnosis since no semi-structured interview was performed. Similarly, childhood trauma information was obtained from a self-report questionnaire, which does not provide a reliable estimation of the presence or absence of childhood trauma [66]. Nevertheless, we could not exclude that participants may have felt uncomfortable disclosing such experiences [19]. Furthermore, CTQ does not provide information about the duration and the severity of each childhood trauma. Second, the diagnosis of mental disorders and substance use disorders were made by a team of different psychiatrists, leading to possible interviewer bias. Third, self-reported data by prisoners about some variables like self-harm, suicide attempts, or the prescription of psychotropic medications may have been unreliable and fragmented. Indeed some prisoners, especially those with personality disorders, can falsify the data to obtain some advantages [67]. Fourth, we separated NSSI from suicide attempts, but we cannot rule out that NSSI and suicide are the same event. Nevertheless, NSSIs and suicide attempts are part of the same continuum in the context of self-harm behaviours [25]. Finally, our study included only male prisoners. This is noteworthy since gender differences were reported for the prevalence of ADHD among prisoners by some authors [68]. However, a meta-analysis of findings from studies about the prevalence of ADHD in detention centres did not find gender differences in the prevalence of the condition [5]. This is divergent from general population studies, which reported a large gender disparity [22]. Moreover, as the sample consisted of information from individuals of a single prison, the results might not be generalisable to all prisoners. Nevertheless, the limited size of the sample may influence the power of the study to draw definitive conclusions. Finally, the small sample size prevents analyses of ADHD subtypes.

## 5. Conclusions

Despite some limitations, the current study shows that ADHD symptoms are common among newly arrived male prisoners. In particular, cocaine may be related to a higher risk of ADHD symptoms than other substances. Moreover, ADHD-P subjects are more likely than the counterpart to have been prescribed mood stabilisers. Therefore, it is likely that ADHD may be overlooked and is often seen as secondary to different apparently more important conditions, such as mood disorders or substance misuse [69]. Of note, one point repeatedly reported in literature is the fear of the distribution of stimulants prescribed to ADHD patients to other prisoners [70]. However, a focus on comorbid disorders may result in a lack of response to treatment if ADHD is not concomitantly managed. On the other hand, proper treatment of ADHD was demonstrated to ameliorate comorbid conditions. In light of these considerations, prisoners affected by ADHD should be promptly identified and properly treated. Even though treatment programs for offenders with ADHD are very limited, targeted psychosocial and pharmacological interventions were reported to reduce recidivism and ideally improve offenders’ quality of life [23]. The reduction of delayed recognition and treatment of prisoners affected by ADHD results are even more significant considering the younger age of prisoners with ADHD than those without this condition and the related societal costs [71,72]. In this regard, even though our findings cannot identify a causal association between childhood trauma and ADHD, the present study recommends exploring the eventual presence of childhood trauma in prisoners to assess vulnerability to ADHD. Further longitudinal studies with larger samples are needed to confirm the results of the present article, perhaps taking into account the three subtypes of the disorder (predominantly inattentive, predominantly hyperactive-impulsive, and combination) [73] and to investigate other environmental or biological factors associated with ADHD. Nevertheless, further studies exploring differences in the gender ratio of ADHD in prison population should be performed. Since in the prison population the ratio of males to females with ADHD seems much reduced compared to general population, it could be interesting to evaluate protective mechanisms/risk factors associated with incarceration among female offenders with ADHD [5]. Future research, therefore, will identify robust clinical and biological predictors of ADHD with the aim to implement prevention strategies and targeted treatments [74].

## Figures and Tables

**Table 1:** Socio-demographic and clinical features of the total sample and of groups divided according to the presence of ADHD symptoms *.

Variables	Total Sample *n* = 108	ADHD-N *n* = 73 (67.6%)	ADHD-P *n* = 35 (32.4%)	*p*-Value
Sociodemographic				
Age (years) *mean* (SD)	36.3 (11.7)	38.0 (11.8)	32.8 (10.9)	0.028 ^a^
Non-Italian nationality	31 (28.7%)	20 (27.4%)	11 (31.4%)	0.665 ^b^
Partner/married	42 (38.9%)	30 (41.1%)	12 (34.3%)	0.497 ^b^
Low-medium level of education **	92 (85.2%)	62 (84.9%)	30 (85.7%)	1.000 ^c^
Unemployed	48 (44.4%)	32 (43.8%)	16 (45.7%)	0.854 ^b^
Any previous incarceration	61 (57.0%)	39 (54.2%)	22 (62.9%)	0.394 ^b^
Diagnosis				
Psychotic disorders	12 (11.2%)	9 (12.3%)	3 (8.8%)	0.749 ^c^
Bipolar disorder	1 (0.9%)	0 (0.0%)	1 (2.9%)	0.324 ^c^
Depressive and adjustment disorder	12 (11.1%)	9 (12.3%)	3 (8.6%)	0.748 ^c^
Personality disorders	47 (43.5%)	26 (35.6%)	21 (60.0%)	0.017 ^b^
Anxiety disorders	4 (3.7%)	3 (4.1%)	1 (2.9%)	1.000 ^c^
Obsessive compulsive disorder	2 (1.8%)	1 (1.4%)	1 (2.9%)	0.545 ^c^
Substance use disorder				
Alcohol	36 (33.3%)	22 (30.1%)	14 (40.0%)	0.309 ^b^
Cannabis	65 (60.2%)	40 (54.8%)	25 (71.4%)	0.098 ^b^
Cocaine	79 (73.1%)	47 (64.4%)	32 (91.4%)	0.003 ^b^
Opioids	22 (25.7%)	13 (17.8%)	9 (25.7%)	0.444 ^c^
Others	18 (16.8%)	11 (15.1%)	7 (20.0%)	0.585 ^c^
NSSI (lifetime)				
In prison	27 (25.0%)	13 (17.8%)	14 (40.0%)	0.013 ^b^
Out of prison	17 (15.7%)	9 (12.3%)	8 (22.9%)	0.170 ^c^
Suicide attempt (lifetime)				
In prison	9 (8.3%)	3 (4.1%)	6 (17.1%)	0.056 ^c^
Out of prison	10 (9.3%)	6 (8.2%)	4 (11.4%)	0.725 ^c^
Outpatient mental health services				
*Child* and adolescent	16 (4.8%)	7 (9.6%)	9 (25.7%)	0.041 ^c^
Adult	30 (27.8%)	18 (24.7%)	12 (34.3%)	0.170 ^b^
Psychotropic medications				
Antipsychotic	38 (35.2%)	25 (34.2%)	13 (37.1%)	0.768 ^b^
Mood stabiliser	32 (29.6%)	12 (16.4%)	20 (57.1%)	0.000 ^b^
Antidepressant	14 (13.0%)	7 (9.6%)	7 (20.0%)	0.219 ^c^
*Anxiolytics or hypnotics*	73 (67.6%)	44 (60.3%)	29 (82.9%)	0.019 ^b^
Comorbid medical conditions	47 (43.5%)	34 (46.6%)	13 (37.1%)	0.355 ^b^
Victimisation				
Emotional abuse	22 (20.4%)	10 (13.7%)	12 (34.3%)	0.013 ^b^
Physical abuse	21 (19.4%)	12 (16.4%)	9 (25.7%)	0.302 ^c^
Sexual abuse	3 (2.8%)	2 (2.7%)	1 (2.9%)	1.000 ^c^
Emotional neglect	15 (13.9%)	7 (9.6%)	8 (22.9%)	0.078 ^c^
Physical neglect	11(10.2%)	4 (5.5%)	7 (20.0%)	0.036 ^c^

Notes Values are numbers (%) with the exception of age. ^a^ *t* test; ^b^ Pearson’s χ^2^ test; ^c^ Fisher’s exact test. * Participants who met the criteria on both Wender Utah rating scale (WURS) and the adult ADHD self-report scale v1.1 (ASRS-V 1.1) were considered to be positive for ADHD. ** Education was dichotomised as low-medium level (primary or secondary school) vs. high level (college or university degree or higher). ADHD = attention-deficit/hyperactivity disorder; ADHD-N = ADHD screened negative; ADHD-P = ADHD screened positive; NSSI = nonsuicidal self-injury; SD = standard deviation.

**Table 2:** Logistic regression analysis for the odds of patients to have positive results for ADHD *.

Variables	aOR	95% CI	*p*-Value
Age	0.97	0.93–1.02	NS
Any previous incarceration	1.22	0.43–3.45	NS
Personality disorders	0.99	0.32–3.03	NS
Cocaine use disorder	5.60	1.30–23.94	*
NSSI in prison	1.43	0.44–4.65	NS
Child and adolescent mental health services	1.96	0.46–8.40	NS
Mood stabilisers	5.14	1.74–15.23	**
Anxiolytics or hypnotics	1.20	0.32–4.51	NS
Emotional abuse	3.65	1.07–12.44	*
Physical neglect	2.23	0.41–12.02	NS

Notes Model adjusted for age and any previous incarceration. * Participants who met the criteria on both the Wender Utah rating scale (WURS) and the adult ADHD self-report scale v1.1 (ASRS-V 1.1) were considered to be positive for ADHD. ADHD = attention-deficit/hyperactivity disorder; aOR = adjusted odds ratios and their 95% confidence interval (CI); NS = not significant; NSSI = nonsuicidal self-injury. * *p* < 0.05; ** *p* < 0.01.

## Data Availability

Data available on request due to restrictions, e.g., privacy or ethical.
